# Intravenous versus subcutaneous delivery of biotherapeutics in IBD: an expert’s and patient’s perspective

**DOI:** 10.1186/s12919-021-00230-7

**Published:** 2021-12-09

**Authors:** Laimas Jonaitis, Srdjan Marković, Klaudia Farkas, Liana Gheorghe, Željko Krznarić, Riina Salupere, Viktorija Mokricka, Zoya Spassova, Dimo Gatev, Isabella Grosu, Anton Lijović, Olga Mitrović, Mateja Saje, Eszter Schafer, Viktor Uršič, Tina Roblek, David Drobne

**Affiliations:** 1 Gastroenterology clinic, Hospital of Lithuanian University of Health Sciences, Eiveniu str. 2, LT-50009 Kaunas, Lithuania; 2 Department of Gastroenterology, University Hospital Medical Center Zvezdara, Dimitrija tucovica 161, Belgrade, 11000 Serbia; 3 Department of Gastroenterology, University of Szeged, Kálvária sgt. 57, Szeged, 6725 Hungary; 4 Center for Digestive Diseases and Liver Transplantation, Fundeni Clinical Institute, Fundeni 258, 022328 Bucharest, Romania; 5 Department of Gastroenterology, University Hospital Center Zagreb, Kišpatićeva 12, 10 000 Zagreb, Croatia; 6 Division of Gastroenterology, Tartu University Hospital, University of Tartu, Ludvig Puusepa 8, 50406 Tartu, Estonia; 7 Pauls Stradiņš Clinical University Hospital, 13 Pilsoņu iela, Riga, LV 1002 Latvia; 8 Clinic of Gastroenterology, University Hospital “St. Ivan Rilski”, 1431 Sofia, Bulgaria; 9 BABKUK Bulgarian patient organization ( Bulgarian Crohn and Ulcerative Colitis Association), Nikolai Kopernik 28-30, 1000 Sofia, Bulgaria; 10 Romanian IBD Patient Association, Traian 3, 910040 Calarasi, Romania; 11 Patient Organization HUCUK (Hrvatsko udruženje za Crohnovu bolest i ulcerozni colitis), Ulica Kralja Zvonimira 20, 10 000 Zagreb, Croatia; 12 Clinic for Gynecology and Obstetrics, University Clinical Centar of Serbia, Koste Todorovica 26, Belgrade, 11000 Serbia; 13 Inflammatory Bowel Disease Association (Društvo za kronično vnetno črevesno bolezen), Ljubljanska ulica 5, 2000 Maribor, Slovenia; 14 Department of Gastroenterology, Military Hospital Budapest, Podmaniczky u. 111, Budapest, 1062 Hungary; 15 Takeda Pharmaceuticals d.o.o, Bleiweisova cesta 30, 1000 Ljubljana, Slovenia; 16 University medical Centre Ljubljana, Zaloška cesta 2, 1000 Ljubljana, Slovenia; 17 Medical Faculty, University of Ljubljana, Vrazov trg 2, 1000 Ljubljana, Slovenia

**Keywords:** Inflammatory bowel disease, Subcutaneous administration, Intravenous administration, Biotherapeutics, Compliance, Quality of life

## Abstract

Several biologic treatments are available in addition to intravenous also in subcutaneous form for treatment of chronic diseases. Benefits of the subcutaneous application of drugs include self-administration by the patient, shorter time of application process with less infusion related adverse events and consequently lower healthcare costs. With appropriate education and support patients are able to administer their treatments at home. This leads to improvement of quality of life, reduction of time needed to travel to the healthcare institution and consequently also reduces costs also for the patient.

Over one million residents in the USA and 2.5 million in Europe are estimated to have inflammatory bowel disease (IBD), with substantial costs for health care. These estimates do not factor in the ‘real’ price of IBD, which can impede career aspirations, instil social stigma and impair quality of life in patients.

The Virtual Community Meeting, which offered an exchange of experience and opinions from healthcare professionals who are active in treating IBD, and patients with this chronic disease, revealed in-depth arguments and answers to some essential questions: which patients prefer subcutaneous over intravenous dosing; which patients continue to favour intravenous infusions; what are the limitations regarding both applications; what is the patient’s role in therapeutical decision-making and how does IBD affect the patient’s work, finances and quality of life? The aim of this article is to discuss the differences between subcutaneous and intravenous dosing from the health-economic, scientific, and personal perspectives.

The meeting offered strong confirmation that most of the patients and healthcare professionals prefer subcutaneous over intravenous drug administration but emphasise the management of risks associated with treatment compliance. Patient education provided by the IBD team in this regard is mandatory. Quality of life of patients is poorer during active disease, but the findings that it can improve over time, including as a result of home- or self-administration of biologics, may be encouraging for individuals with this chronic disease.

## Introduction

Inflammatory bowel disease (IBD) is a chronic disease which can cause progressive functional and structural damage to the gastrointestinal tract. IBD comprises two types: ulcerative colitis (UC) and Crohn’s disease (CD). It was traditionally regarded as a disease of the westernised nations, but in the twenty-first century, the epidemiology of IBD has been changing fast [1]. Within Europe, the highest incidence and prevalence rates are found in Scandinavia and the United Kingdom while the diseases, according to the latest data available, remain rare in Eastern Europe. However, the occurrence of IBD is a dynamic process as demonstrated by the increasing incidence rates being reported from previously low incidence areas, including Eastern Europe. The incidence of CD in Europe ranges from 0.5 to 10.6 cases per 100,000 person-years while the estimates for UC range from 0.9 to 24.3 per 100,000 person-years [2].

Over the last decade, biologics have gained an important place in the treatment of moderate to severe IBD, and many randomised control trials have evaluated their efficacy. Currently, monoclonal antibodies against tumour necrosis factor-alpha (infliximab, adalimumab, certolizumab, and golimumab), integrins (vedolizumab and natalizumab*), and interleukin (IL)-12 and IL-23 antagonists (ustekinumab) are approved for use in IBD [3].

Subcutaneous delivery of biotherapeutics has become a valuable alternative to intravenous administration across many disease areas. Subcutaneous administration has been proven to be effective, safe, well-tolerated, generally preferred by patients and healthcare providers, and it results in reduced drug delivery-related healthcare costs and resource use [3]. The aim of this report is to present the results from the Virtual Community Meeting which took place from January 21 to February 4, 2021 and contributed relevant insights regarding intravenous and subcutaneous drug administration from healthcare professionals located in nine Central and Eastern European Countries (Bulgaria, Croatia, Estonia, Hungary, Latvia, Lithuania, Romania, Serbia, Slovenia) and patient organisation representatives from six Central and Eastern European Countries (Bulgaria, Croatia, Hungary, Romania, Serbia, Slovenia). As IBD is a chronic disease, involving patients in the treatment decision-making process is very important.

## Intravenous versus subcutaneous drug administration: an expert’s perspective

Because subcutaneous administration allows self-injections outside the hospital setting, it reduces patient dependency on hospital facilities and results in reduced drug delivery-related healthcare costs and resource use. It is also less time-consuming and minimises the discomfort associated with intravenous infusion. Adverse events associated with subcutaneous administration may occur due to inappropriate storage of the drug at home or potential local allergic manifestations. According to the Virtual Community Meeting, the COVID-19 pandemic did not influence the administration of biologics, but it is evidently easier to manage subcutaneous formulation during the pandemic.

The main drivers for switching from intravenous to subcutaneous administration of biologics can be divided into three groups: medical considerations (disease improvement/stabilisation, facility decongestion, patient involvement in management), patient considerations (preference for a more comfortable and easy-to-deliver formula, self-administration, a more flexible schedule, a limited dependence on medical facilities and staff), and administrative considerations involving costs and, in some countries, insurance reimbursement.

Participating experts agreed that almost all patients would be suitable for a subcutaneous formulation as maintenance therapy. There are, however, concerns associated with self-administration in patients with a low educational level, and with non-compliant patients. Some patients find it difficult to overcome their fear of self-injecting. Some patients find it too frequent to administer an injection every week or every second week, while the intravenous infusion is usually needed just once in 8 weeks. Many patients also feel safer if they receive the medication intravenously in a hospital because they trust the medical staff and they may feel that their disease is more under control if the treatment process is left to the professionals. To overcome these psychological and objective barriers, extended patient education and open communication in this regard is mandatory.

**Fig. 1:**
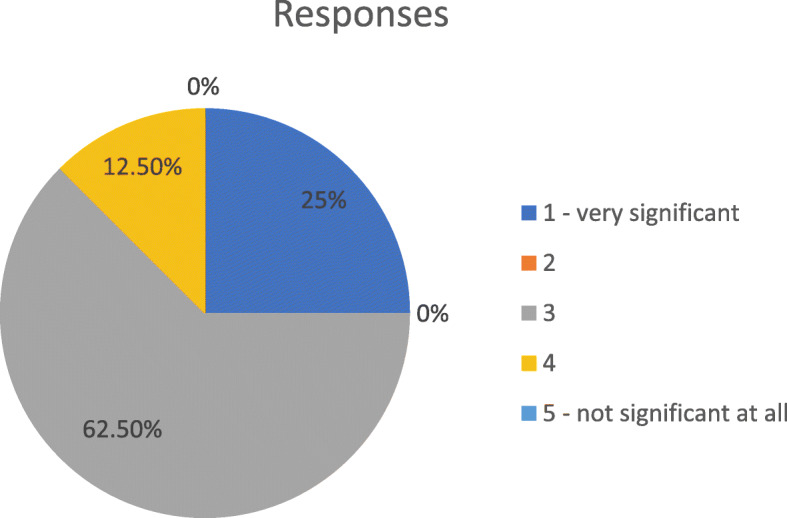
Scale of importance of patients’ role in the choice of biologic formulation

Among the patients suitable for subcutaneous administration of biologics, experts assessed the proportion of those suitable for injector pen/auto-injector pen versus pre-filled syringe: the majority agreed that 80 to 95% of their patients prefer injector pen/auto-injector pen as a delivery method. The importance of patients’ role in the choice of biologic formulation is presented in are presented in Fig. 1.

## Compliance/adherence risks and additional medical support

Patient education provided by the IBD team (a nurse and a physician) is most important for correct self-injecting practice. Patients must be trained, and their skills assessed after the training. Data regarding the patient’s experience must be collected during the treatment and reasonable control of their skills must also be performed. Additional tools that could help the patients are, for example, training kits, leaflets, and simple video instructions. During the COVID-19 pandemic, mandatory social distancing and a lack of effective treatments have made telemedicine the safest interactive system between patients, both infected and uninfected, and clinicians.

Patients support programmes (such as IBD nurse support of patients and calls to remind them to take their medication) could be very useful for IBD patients, but they are underdeveloped or non-existent in most of the countries involved in the Virtual Community. The use of mobile applications to monitor/manage the disease is not common in IBD practices, although researchers and practitioners are developing these tools to provide better monitoring of their patients and to improve compliance. The main compliance/adherence risks are incorrect administration of injections, missed doses, and inappropriate storage, which raises the need for a steady temperature control.

## Age, education, and history of the disease

Taking into account all the circumstances, HCPs discussed which patients would be more suitable for intravenous and which for subcutaneous application. Patients with IBD who have a long history of disease, patients with more severe and active disease, and patients with a history of previous unsuccessful treatment with other biologics are more suitable candidates for intravenous medication. The same applies to less educated patients and patients with a fear of self-injections. On the other hand, young patients who have a short history of disease which is under control after induction therapy and who have no reservations about self-injections are potential candidates for maintenance subcutaneous application. These are mainly educated, active people. The participants of the meeting had already accumulated a considerable amount of experience with vedolizumab intravenous therapy. Most of them would initiate a maintenance regimen with subcutaneous administration of the drug as soon as it is available on the market if the disease is well controlled – when remission is achieved.

**Fig. 2:**
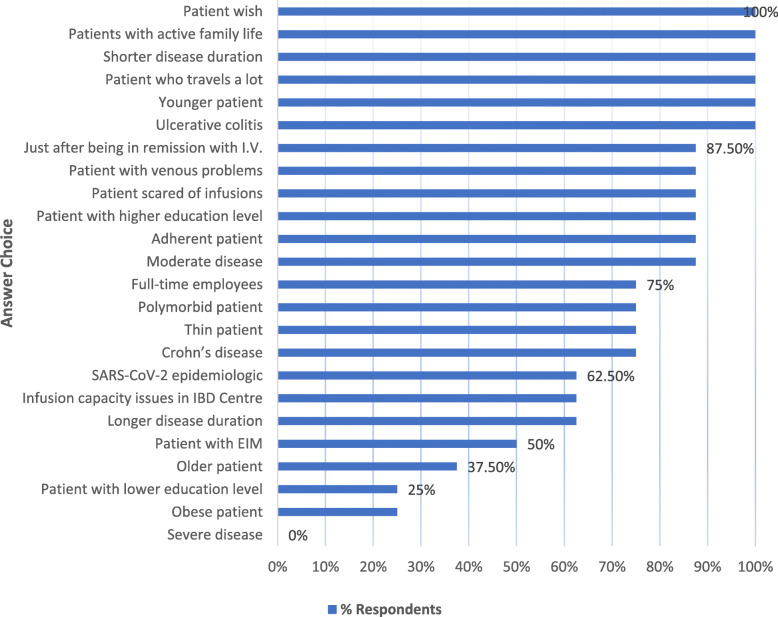
Factors impacting favorable choice of subcutaneous application

The main factors impacting the choice of subcutaneous application are presented in Fig. 2.

## Intravenous versus subcutaneous drug administration: a patient’s perspective

At the Virtual Community Meeting, six patient representatives all expressed preference for subcutaneous administration and presented a long list of positive experiences. It is less time-consuming, and it takes less effort and time absent from work when self-administration is performed at home, they reported. Subcutaneous application systems are designed with smaller needle sizes, which may decrease pain during administration. Furthermore, administration at home reduces the risk of exposure to hospital-acquired infections. Subcutaneous application is expected to improve patient quality of life and provide support to patients who live far from a hospital or have difficulties travelling and parking in the vicinity of the hospital. This can contribute to a lower financial burden, taking into consideration that IBD patients often have part-time jobs or are unemployed. Nevertheless, visiting the hospital is time-consuming and is often a burden for another family member or friend who accompanies the patient.

Patients – as with health care professionals – are convinced that the subcutaneous route of application is more suitable for younger, employed patients, while the intravenous route is more appropriate for older patients, especially those who refuse to inject themselves and feel safer when receiving therapy in a hospital environment.

## Convenience of choice and less frequent administrations

**Fig. 3:**
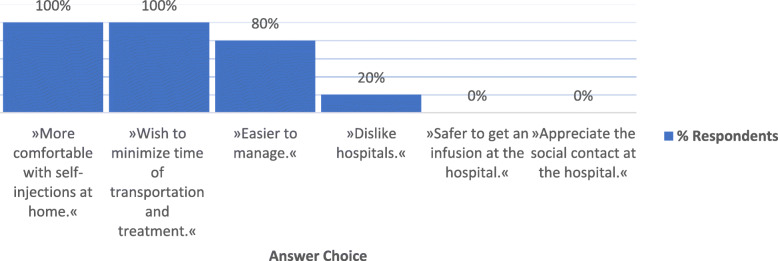
Main reasons for patients” preference of the preferring subcutaneous route of application

Which kind of drug administration would patients prefer? Oral - if this is not available, then subcutaneous, as reported by the patient representatives. The most important decisive factors are less frequent drug administrations and the convenience of choice. As IBD is a chronic disease, involving patients in the decision-making process is very important. The patient should know the benefits, risks, and adverse reactions associated with the treatment. The decision on the most appropriate biological therapy should be made by the patient and the doctor together. The main reasons for patinets to prefer subcutaneous application are presented in Fig. 3.

## Quality of life and the patient’s role

Numerous studies have shown that health-related quality of life is impaired in patients living with IBD as compared with the general population. While disease activity and severity are an important driver of physical and mental health-related quality of life, patients may experience psychological distress even during clinical remission. Living with IBD can impact employment, family planning, and personal milestones. Furthermore, the impact of IBD extends to the patient influencing the quality of the lives of those around them, including their caregivers [4].

The vision of IBD patient societies is to provide assistance, patient counselling, education, and verified information and knowledge about the disease. It is not enough that only the members of the IBD society are educated. As the disease affects all aspects of the patient’s life, it is important that the professional and the general public are well-informed, too, and that they can recognise the patient’s needs and make the appropriate adjustments for their well-being. Recently, patient societies have become involved in scientific and therapeutic activism. The concept of the ‘expert patient’ or the ‘expert of experience’ has developed. Many patient organisations have developed processes and methodologies to ensure that their members are fully prepared to become involved in areas such as research and clinical trials.

## Conclusions

The Virtual Community Meeting delivered some interesting findings about the treatment of IBD based on the view of healthcare professionals and patient organisation representatives from Bulgaria, Croatia, Estonia, Hungary, Latvia, Lithuania, Romania, Serbia, and Slovenia. Preference studies and practical experience have revealed that most of the patients and healthcare professionals prefer subcutaneous over intravenous drug administration. Among the patients suitable for subcutaneous administration of biologics, 80 to 95% prefer injector pen/auto-injector pen as a delivery method. Patient education provided by the IBD team (a nurse and a physician) is most important for correct self-injecting practice and management of the risks associated with treatment compliance (wrong injection applications, missed doses, inappropriate storage of the drug). As IBD is a chronic disease, involving patients in the therapy decision-making process is very important. Patient education and their involvement in the decision-making process increases their responsibility and treatment compliance, which have an important impact on the efficiency of the disease management.

## Data Availability

All the transcripts of the questions, insights and comments are available at Takeda Pharmaceuticals d.o.o. and by the corresponding author.

## Abbreviations

IBD: Inflammatory bowel disease
UC: Ulcerative Colitis
CD: Crohn’s disease
IL: Interleukin
HCP: Healthcare professionals
SC: Subcutaneous
IV: Intravenous
